# Identified Candidate Genes of Semen Trait in Three Pig Breeds Through Weighted GWAS and Multi-Tissue Transcriptome Analysis

**DOI:** 10.3390/ani15030438

**Published:** 2025-02-05

**Authors:** Xiaoke Zhang, Zhiting Xu, Qing Lin, Yahui Gao, Xiaotian Qiu, Jiaqi Li, Shuihua Xie

**Affiliations:** 1 State Key Laboratory of Swine and Poultry Breeding Industry, National Engineering Research Center for Breeding Swine Industry, Guangdong Provincial Key Lab of Agro-Animal Genomics and Molecular Breeding, College of Animal Science, South China Agricultural University, Guangzhou 510642, China; zxkstar@163.com (X.Z.); zhitingxu@126.com (Z.X.); qing_lin1996@126.com (Q.L.); gyhalvin@gmail.com (Y.G.); jqli@scau.edu.cn (J.L.); 2 State Key Laboratory of Animal Biotech Breeding, Institute of Animal Science, Chinese Academy of Agricultural Sciences, Beijing 100193, China; 3 National Animal Husbandry Service, Beijing 100125, China; qxt-nahs@agri.gov.cn; 4 Agriculture Technology Extension Centre of Guangdong Province, Guangzhou 510520, China

**Keywords:** pig, semen traits, weighted GWAS, multi-tissue transcriptome analysis, candidate genes

## Abstract

High-quality semen is an essential factor for the success of artificial insemination, and revealing the genetic structure of pig semen traits helps improve semen quality. This study aimed to identify candidate genes associated with semen traits in three pig breeds (Duroc, Landrace, and Yorkshire) through weighted GWAS and multi-tissue transcriptome analysis. In this study, to identify candidate genes associated with semen traits in Duroc, Landrace, and Yorkshire, we performed weighted GWAS in four traits (sperm motility, sperm progressive motility, sperm abnormality rate, and total sperm count) using 936 pigs and multi-tissue transcriptome analysis using 34 tissues RNA-seq data of 5457 pigs from FarmGTEx. It was found that 16, 9, and 12 significant SNPs associated with semen traits were identified in Duroc, Landrace, and Yorkshire, with corresponding 7, 5, and 7 candidate genes in these three breeds, respectively, which may be involved in mammal spermatogenesis, testicular function, and male fertility. Moreover, we not only found the same candidate gene *DNAI2* as in previous studies but also found two new candidate genes *PNLDC1* and *RSPH3*, which were identified simultaneously in both Landrace and Yorkshire. By integrating the GWAS and multi-tissue transcriptome analysis results, we found that candidate genes associated with semen traits of three pig breeds were highly expressed in the testis tissue. The three genotypes of rs320928244 had significant effects on the expression of the *DYNLT1* gene in the testis tissue of Landrace. These results together showed that these candidate genes were mainly related to sperm motility defects. This study helps deepen the understanding of the genetic basis of semen traits and provides a theoretical foundation for improving the semen quality of Duroc, Landrace, and Yorkshire breeds.

## Simple Summary

This study utilized data from 936 pigs for genome-wide association studies (GWAS) of four semen traits (sperm motility, sperm progressive motility, sperm abnormality rate, and total sperm count), as well as data from 5457 pigs from FarmGTEx for transcriptome analysis. The results revealed 16, 9, and 12 significant single nucleotide polymorphisms (SNPs) associated with semen traits in Duroc, Landrace, and Yorkshire pigs, with 7, 5, and 7 candidate genes identified in these breeds, potentially linked to mammalian spermatogenesis, testicular function, and male fertility. This research deepened the understanding of semen trait genetics and provided insights for enhancing semen quality in these pig breeds.

## 1. Introduction

With the rapid development of artificial insemination (AI), it has become important to select boars with outstanding semen quality to improve fertilization outcomes [1]. Traditional breeding schemes have focused on selecting pigs with excellent growth performance, carcass performance, and female reproductive traits while neglecting the potential benefits of semen traits on reproductive performance [2]. In recent years, with the rapid development of high-throughput genotyping and molecular techniques, genetic markers have gradually been utilized in genetic evaluation. There has been increasing interest in studying the molecular processes and genetic mechanisms that affect semen traits [3]. By revealing the genetic structure and candidate genes associated with pig semen traits, the efficiency of genome selection for target traits can be improved.

Genome-wide association studies (GWASs) commonly use high-throughput genotyping technology to assay single nucleotide polymorphisms (SNPs) and associate them with traits of interest [4]. At present, several studies have reported candidate genes and markers associated with pig semen traits by GWAS. Diniz et al. [5] reported the *MTFMT* gene associated with sperm motility traits in Landrace. Marques et al. [6] reported six/five candidate genes associated with semen traits in Landrace/Yorkshire. Godia et al. [7] identify candidate genes associated with semen traits and a series of mRNAs linked to sperm biology in Pietrain pigs by genome and transcriptome data. Additionally, Gao et al. [8], Zhao et al. [9], Mei et al. [10], and Zhang et al. [2] reported some candidate genes associated with semen traits in Duroc.

Previous GWAS studies in pig semen traits were primarily performed using traditional single SNPs GWAS and weighted single-step GWAS. Weighted single-step GWAS illustrates genetic variation through windows instead of single SNPs [6,11]. Because of the importance of semen quality in pig breeding, understanding the significant effects of single SNPs is crucial to designing selection programs. The weighted GWAS, proposed by Li et al. [12], is a method that assigns weights to residual variances, aiming to reduce stratification and stabilize solutions in GWAS. The weighted GWAS may outperform traditional GWAS methods when the number of animals with both phenotypes and genotypes is small. This method has been successfully applied to milk production traits [12]. In GWAS, the dependent variable must be a single value, whereas de-regressed estimated breeding values (DEBVs) combine repeated measures into a single value. DEBVs have proven to be effective response variables in GWAS studies of cattle milk production [13] and pig semen traits [10].

The integration of GWAS and multi-tissue transcriptome analysis helps understand the genetic structure of complex traits in cattle [14] and pigs [15]. To date, few studies have used genetics and transcriptome information to study the genetic background of pig semen traits. Godia et al. [7] identified three trans-expression QTL involving the candidate genes through the integration of GWAS (288 pigs) and sperm RNA sequencing (RNA-seq) data (35 pigs) analysis. The integration of GWAS and multi-tissue transcriptome analysis can provide a more intuitive understanding of the expression and biological significance of candidate genes in pig tissues.

In this study, we performed weighted GWAS using 936 pigs and multi-tissue transcriptome analysis using 34 tissues with 5457 RNA-seq data from FarmGTEx (http://piggtex.farmgtex.org/, accessed on 8 April 2024). The aim was to identify genetic variants and candidate genes associated with semen traits in three pig breeds. Subsequently, we analyzed the gene expression levels of candidate genes that could have the most significant impact on spermatogenesis and male fertility in 34 pig tissues. To gain a deeper understanding of the genetic mechanisms underlying pig semen traits, we performed a post-GWAS analysis that involved Gene Ontology (GO) and the Kyoto Encyclopedia of Genes and Genomes (KEGG). This analysis helps us to understand the biological processes and functional annotations of the candidate genes.

## 2. Material and Methods

### 2.1. Population and Phenotype Data

From August 2022 to February 2024, a total of 28,321 semen records were collected from 936 boars (382 Duroc, 290 Landrace, and 264 Yorkshire) at one artificial insemination station (Guangdong Guyue Technology Co., Ltd., Guangzhou, China). The boar populations were reared in individual fences with ad libitum access to drinking water, 14 h of daily light, and a daily diet of 2.5 kg, with adjustments made for individual boars based on their actual feed intake. Semen was collected every morning. Boars were introduced to the artificial insemination station from the same breeding farm at 4 months of age. These individuals shared similar genetic backgrounds, ages, and rearing conditions, which ensured consistency in evaluating semen traits and minimized external influences such as age differences or environmental stress. The pedigree file included 961 individual animals and four-generation pedigree records. The age of ejaculation for the boars ranged from 6.47 to 23.27 months. Four semen traits were measured, including sperm motility (SPMOT), sperm progressive motility (SPPMOT), sperm abnormality rate (SPABR), and total sperm count (SPCOUNT). After the semen was collected, it was immediately stored in an insulated bucket and kept at a temperature of 37 ± 1 °C, using a water bath in the laboratory. Subsequently, the SPMOT, SPPMOT, and SPABR of the original semen were measured using the Spain MagaporGesipor3.0 CASA system (CASA system analyzes SPMOT by tracking the movement of individual sperm cells in the sample, which refers to the proportion of motile sperm cells to the total number of sperm. SPPMOT: the proportion of sperm cells that move in a straight line. SPABR: the proportion of sperm cells with abnormal morphology. SPCOUNT: the total number of sperm cells in semen). The process of measuring semen traits typically takes approximately 10 min from the collection of the original semen. The SPCOUNT was calculated by multiplying the semen volume (mL) by the semen concentration (10^6^/mL). The phenotypic values of semen traits were multiply compared using the “agricolae” package of R (v4.0.3) software.

The summary statistics of pig semen traits for three pig breeds are shown in Table 1. Referring to the research of Marques et al. [16] and Wang et al. [17], the quality control of phenotypes was as follows: (1) semen records with semen collection times less than 5 were excluded; (2) semen records with semen volume ≤ 50 mL were removed; (3) semen records with sperm motility < 10% were removed; (4) semen records with adjacent semen collection interval > 60 days or equal to 0 days were removed.

### 2.2. Genotype Data

Genomic DNA was extracted and purified from semen samples (The semen samples were centrifuged at low speed to collect sperm) of 850 pigs, which included 361 Duroc, 257 Landrace, and 232 Yorkshire, using the TIANcombi DNA Lyse&Det PCR Kit (TIANGEN Biotech Co., Ltd. in Beijing, China). These pigs were genotyped using the 50 k SNP array (KPS Porcine Breeding Chip v1, Beijing, China), which contained 57,466 SNPs. We removed 12,213 SNPs that were not mapped to the reference genome (*Sus scrofa 11.1*). To detect outliers, we performed principal components (PCs) analysis on the genotype data using the R package Sommer [18]. For each breed, we excluded SNPs with a call rate lower than 0.9, a minor allele frequency (MAF) lower than 0.01, and a Hardy–Weinberg equilibrium (HWE) lower than 1 × 10^−6^ using PLINK v1.90 [19]. Finally, we retained 31,618 SNPs for Duroc (361 pigs), 33,704 SNPs for Landrace (257 pigs), and 39,925 SNPs for Yorkshire (232 pigs), respectively.

### 2.3. Variance Component Estimation and DEBV Calculation

The variance components of semen traits for each breed were computed using the average information restricted maximum likelihood (AI-REML) by DMU (version 6-R5-2-EM64T) software [20]. The mixed linear model was as follows:

(1) y=μ+Xf+Za+Wp+Age+Intv+e

where y is the vector of phenotypes; μ is the vector of phenotype means; f is the vector of fixed effects (overall mean year-season of ejaculation and birth parity of boar, these fixed effects had significant effects on semen quality of boars by *F*-test); a~*N*(**0**,$A\sigma_{a}^{2}$) is the vector of additive genetic effects, $\sigma_{a}^{2}$ is the additive genetic variance, A is the pedigree matrices; p~*N*(**0**,**I** $\sigma_{p}^{2}$) is the vector of permanent environment effects; $\sigma_{p}^{2}$ is the variance of permanent environmental effects; X, Z and W are the design matrix corresponding to f, a**,** and p, respectively. The covariates Age and Intv represent the months of age and the intervals between ejaculations, respectively. e~*N*(**0**, **I**$\sigma_{e}^{2}$) is the residual effect vector, $\sigma_{e}^{2}$ is the residual variance, I is the identity matrix.

DEBVs were calculated according to VanRaden [21]. The formulas are described as follows:

(2) $${DEBV}_{i}=PA+\frac{{EBV}_{i}-PA}{R_{i}}$$

(3) $$PA=\frac{{EBV}_{s}+{EBV}_{d}}{2},R_{i}=\frac{{DE}_{i}-{DE}_{PA}}{{DE}_{i}}$$

(4) $${DE}_{i}=k\frac{{REL}_{i}}{1-{REL}_{i}},{REL}_{PA}=\frac{{REL}_{s}+{REL}_{d}}{4},k=(1-h^{2})/h^{2}$$

where DE is the daughter equivalents; REL is the reliability of EBV; $h^{2}$ is the heritability of semen traits; i, s, d, and PA are individual animal, sire, dam, and parent average, respectively.

### 2.4. Weighted Genome-Wide Association Study

Single trait-weighted GWAS was carried out by a mixed-model approach using MMAP (mmap.2021_08_19_22_30.intel, 2021) [22] software. The mixed model is as follows:

(5) y=Xb+g+e

where y is the vector of DEBVs for each semen trait; **X** is a matrix of genotypes (with 0, 1, or 2 represented genotypes AA, Aa, and aa, respectively), and b is a vector of marker effects; **g**~*N*(**0**, $G\sigma_{g}^{2}$) is the vector of polygenic effects accounting for population structure, where G is the genomic relationship matrix built by using all markers and $\sigma_{g}^{2}$ is the genetic variance; and $e\sim N(0,R\sigma_{e}^{2})$ is the vector for residual effect, where $\sigma_{e}^{2}$ is residual variances, **R** is a diagonal matrix weighted by the reliability of DEBVs and heritability.

Li et al. [12] demonstrated that considering the residual variances in the weighting process can effectively minimize stratification and enhance the stability of the solution. The weights utilized in **R** were associated with the reliability of conventional DEBVs (${Rel}_{DEBV}$) and the reliabilities of traditional PA [12]:

(6) $${Weight}_{Animal}=\frac{1-h^{2}}{\left[c+(1-{Rel}_{Animal})/{Rel}_{Animal}\right]h^{2}}$$

where $h^{2}$ is the heritability for each trait; c is the proportion of genetic variation that cannot be explained by SNPs, when c = 0.1, the DEBVs have high reliability; ${Rel}_{Animal}$ was computed as a function of daughter equivalents (DE):

(7) $${Rel}_{Animal}=\frac{{DE}_{DEBV}-{DE}_{PA}}{{DE}_{DEBV}-{DE}_{PA}+1}$$

and daughter equivalents for DEBV and PA were calculated as:

(8) $${DE}_{DEBV}=\frac{{Rel}_{DEBV}}{1-{Rel}_{DEBV}},\;{DE}_{PA}=\frac{{Rel}_{PA}}{1-{Rel}_{PA}}$$

The genomic inflation factor (λ) was calculated by R software [23] from the raw *p*-values of the GWAS results [24]. The genome-wide false discovery rate (FDR) was applied to avoid false positives caused by multiple tests. The FDR was computed using the R package qvalue (v2.30.0, https://github.com/StoreyLab/qvalue, accessed on 16 March 2024). The threshold for significant SNPs was defined as FDR < 0.05. Manhattan plots and Quantile–Quantile (QQ) plots were performed using the R program. The network analysis and pathway diagrams of candidate genes were drawn using the R package clusterProfiler [25].

### 2.5. Gene Annotation and Functional Enrichment Analysis

The GWAS regions were identified based on the Sus scrofa 11.1 (release 106) database available at Ensembl (https://asia.ensembl.org/Sus_scrofa/Info/Index, accessed on 25 March 2024) by screening a distance of 1 Mb around the significant SNPs, referring to Hering et al. [26]. The function of candidate genes within the GWAS regions was manually identified at the National Center for Biotechnology Information (NCBI, http://www.ncbi.nlm.nih.gov, accessed on 2 April 2024). The Database for Annotation, Visualization, and Integrated Discovery (DAVID, https://david.ncifcrf.gov/tools.jsp, accessed on 6 April 2024) was used to perform the GO and KEGG enrichment analysis of candidate genes.

### 2.6. Integrative of GWAS Results and RNA-Seq Data

Genome and RNA-seq data from 34 tissues (5457 pigs) were downloaded from the Pig Genotype-Tissue Expression (http://piggtex.farmgtex.org/, accessed on 8 April 2024). In short, RNA-seq data were used to calculate gene expression levels (Transcripts per million, TPM) in 34 tissues. GWAS was then performed using gene expression levels as response variables and genomic data to identify expression quantitative trait locus (eQTL). By integrating the GWAS and eQTL results, we further investigated whether the significant SNPs affected gene expression in specific tissues. We recorded the genotype data (the 0, 1, and 2 represented genotypes AA, Aa, and aa, respectively) and then performed multiple comparisons using the least significant difference method based on the three genotypes of the significant SNPs and the expression levels of candidate genes. The LD between the significant SNPs of GWAS results and the significant eQTL of RNA-seq results was assessed using PLINK v1.9 [19] with the default parameters (“--ld-snp” and “--ld-window-kb”). The code for calculating TPM is available at the FarmGTEx Github website (https://github.com/FarmGTEx/PigGTEx-Pipeline-v0, accessed on 8 April 2024).

## 3. Results

### 3.1. Phenotypic Distribution and Variance Components Estimation

The average semen collection times for Duroc, Landrace, and Yorkshire were 37.12 ± 13.38, 27.54 ± 16.02, and 23.31 ± 16.48 times, respectively (Figure S1). The descriptive statistics and heritabilities of four semen traits are shown in Table 1. These findings suggest that the semen traits of Duroc, Landrace, and Yorkshire are moderately heritabilities, with estimations ranging from 0.10 to 0.35. Notably, the heritabilities of semen traits in Duroc were generally higher than those in Landrace and Yorkshire, except for SPPMOT. Table S1 provides more detailed information on the estimation of variance components for semen traits in each breed, including additive variance, permanent environmental effect variance, and residual variance. The distributions of DEBVs for semen traits in the three breeds are shown in Table S2 and Figure S2, indicating a normal distribution.

### 3.2. Weight Genome-Wide Association Study and Mining Candidate Genes

**Figure 1:**
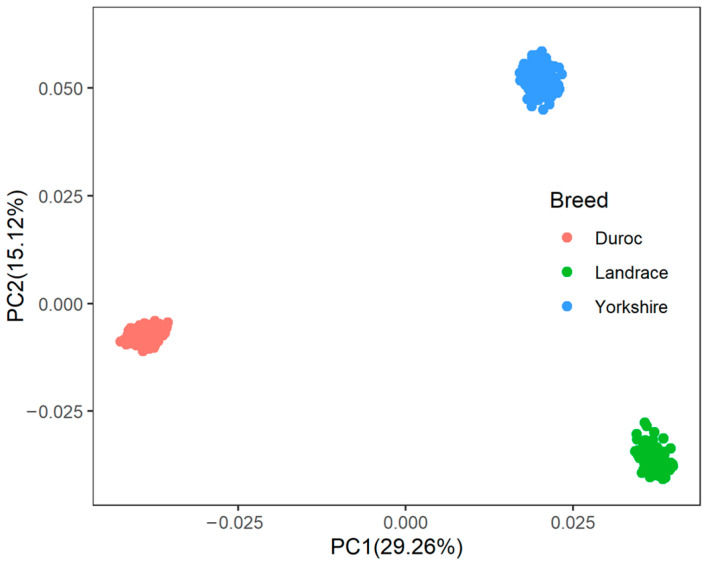
The population structure of three pig breeds. PC1 = the first principal component, PC2 = the second principal component.

A principal component analysis was performed based on the genotypes of each breed (Figure 1). The first two genotype principal components (PCs) explained 29.26% and 15.12% of the total variance, respectively. Table S3 shows all significant SNPs and candidate genes associated with semen traits in the three pig breeds. Specifically, a total of 16, 9, and 12 significant SNPs, as well as 136, 97, and 152 candidate genes, were associated with semen traits in Duroc, Landrace, and Yorkshire, respectively. Table 2 shows promising candidate genes associated with semen traits in three pig breeds, which may be involved in spermiogenesis, and testes functioning. The table also provides details of the significant SNPs. The MAF distributions of the significant SNPs ranged from 0.15 to 0.36. The genomic inflation factors (λ) for four semen traits were close to 1, ranging from 0.89 to 1.09 (Figure S3). The candidate genes *RSPH3* and *DYNLT1*, which were associated with the SPCOUNT trait in Landrace and the SPMOT trait in Yorkshire, were identified in the same region (8.24~8.56 Mb on chromosome 1).

**Figure 2:**
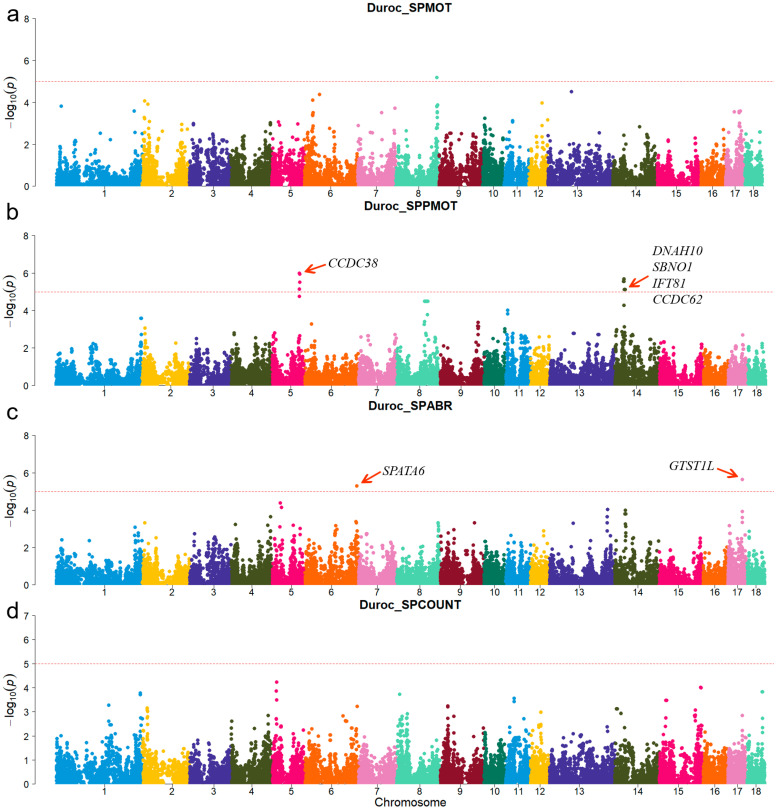
Manhattan plots of semen-trait GWAS results in Duroc pigs. (**a**–**d**) are the SPMOT, SPPMOT, SPABR, and SPCOUNT, respectively.

**Figure 3:**
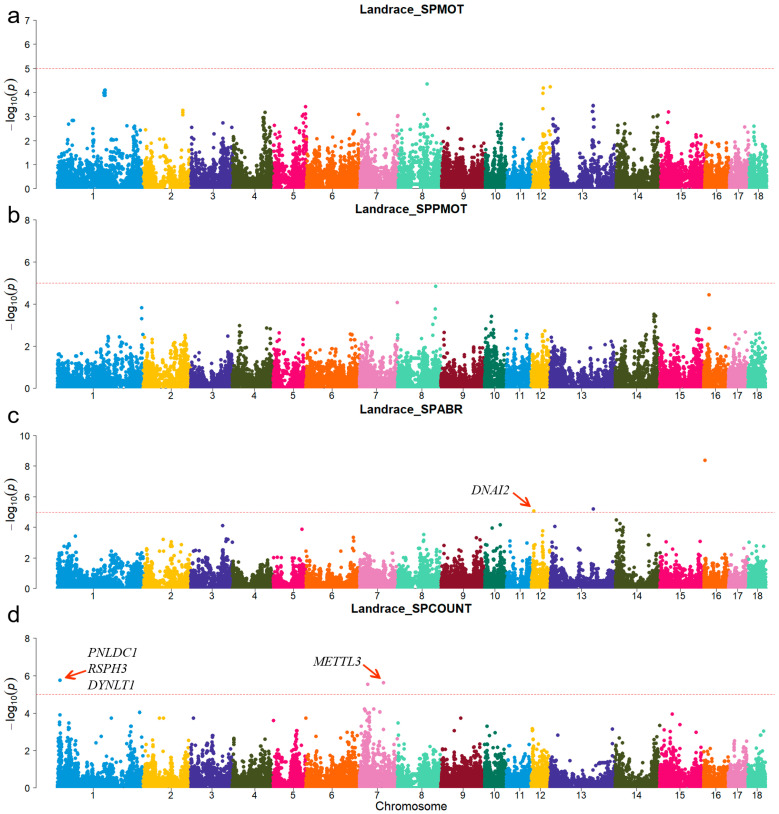
Manhattan plots of semen-trait GWAS results in Landrace pigs. (**a**–**d**) are the SPMOT, SPPMOT, SPABR, and SPCOUNT, respectively.

**Figure 4:**
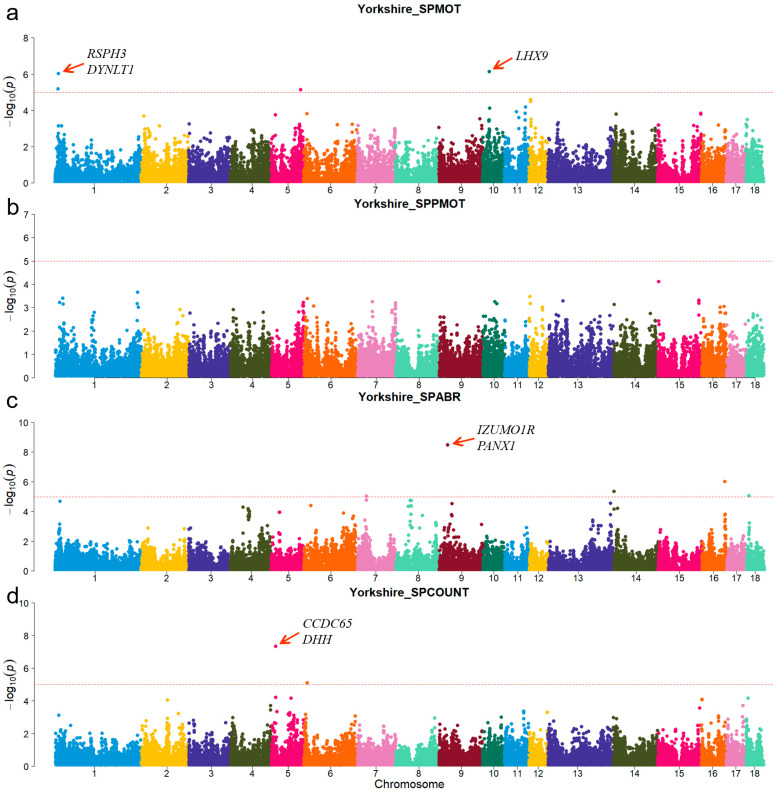
Manhattan plots of semen-trait GWAS results in Yorkshire pigs. (**a**–**d**) are the SPMOT, SPPMOT, SPABR, and SPCOUNT, respectively.

For Duroc, candidate genes coiled-coil domain containing 38 (*CCDC38*), Dynein axonemal heavy chain 10 (*DNAH10*), strawberry notch homolog 1 (*SBNO1*), coiled-coil domain containing 62 (*CCDC62*), and intraflagellar transport 81 (*IFT81*) were identified for the SPPMOT trait, the candidate genes spermatogenesis associated 6 (*SPATA6*) and gametocyte-specific factor 1 like (*GTSF1L*) were identified for the SPABR trait (Table 2 and Figure 2). For Landrace, candidate gene dynein axonemal intermediate chain 2 (*DNAI2*) was identified for the SPABR trait, the candidate genes methyltransferase 3, N6-adenosine-methyltransferase complex catalytic subunit (*METTL3*), PARN-like ribonuclease domain containing exonuclease 1 (*PNLDC1*), radial spoke head 3 (*RSPH3*), and dynein light chain Tctex-type 1 (*DYNLT1*) were identified for the SPCOUNT trait (Table 2 and Figure 3). For Yorkshire, candidate genes *RSPH3*, *DYNLT1*, and LIM homeobox 9 (*LHX9*) were identified for the SPMOT trait, the candidate genes IZUMO1 receptor, JUNO (*IZUMO1R*) and pannexin 1 (*PANX1*) were identified for the SPABR trait, the candidate genes desert hedgehog signaling molecule (*DHH*) and coiled-coil domain containing 65 (*CCDC65*) were identified for the SPCOUNT trait (Table 2 and Figure 4). These candidate genes have shown promising associated with pig semen traits according to the NCBI annotation.

### 3.3. Functional Enrichment Analyses of Candidate Genes

Table 3 shows the enriched GO terms and KEGG pathways of all candidate genes associated with semen traits in three pig breeds. These biological pathways include centrosome (GO:0005813), centriole (GO:0005814), smoothened signaling pathway (GO:0007224), axoneme (GO:0005930), peptidase activity (GO:0008233), ciliary basal body (GO:0036064), cilium assembly (GO:0060271), cilium (GO:0005929), fusion of sperm to egg plasma membrane (GO:0007342), and amyotrophic lateral sclerosis (ssc05014). The candidate genes *DNAH10* (Duroc), *DNAI2* (Landrace), and *CCDC65* (Yorkshire) are involved in the biological process of the axoneme. *CCDC38* and *IFT81* (Duroc) are involved in the biological process of centrosomes. *IFT81* (Duroc) and *CCDC65* (Yorkshire) are involved in the biological process of *the* ciliary basal body. *DNAH10* (Duroc) and *DNAI2* (Landrace) are involved in the biological process of amyotrophic lateral sclerosis.

### 3.4. GWAS and Transcriptome Co-Localization Analysis

**Figure 5:**
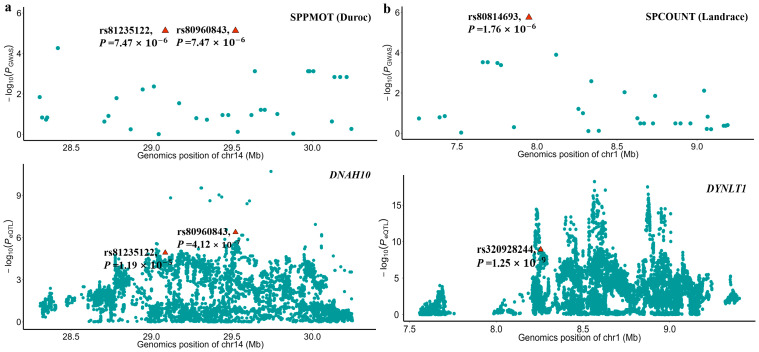
Local Manhattan plots of GWAS and eQTL results. (**a**) The top local Manhattan plot depicted the GWAS of SPPMOT, highlighting the significant SNPs rs81235122 and rs80960843. The bottom local Manhattan plot shows the eQTL mapping of DNAH10 for the pig testis tissue. (**b**) The top local Manhattan plot depicted the GWAS of SPCOUNT, highlighting the significant SNPs rs80814693. The bottom local Manhattan plot shows that the SNP rs320928244 was the eQTL mapping of DYNLT1 in pig testis tissue. The SNP rs320928244 has a high LD (R^2^ = 0.929) with the significant SNP rs80814693. The −log10 *p*-values are shown on the *y*-axis.

The sample sizes of RNA-seq data for each tissue were shown in Figure S4 and ranged from 43 to 1281. By integrating the GWAS and the eQTL results, we found that the significant SNPs, rs80960843 and rs81235122 of SPPMOT, were also identified as eQTL of the *DNAH10* gene in testis tissue. This indicates that the two significant SNPs might affect the phenotype by regulating the expression of the *DNAH10* gene. Additionally, the SNP rs320928244, which has a high LD (R^2^ = 0.929) with the significant SNP rs80814693 of SPCOUNT in Landrace, played a significant role in regulating the expression of the *DYNLT1* gene in testis tissue (Figure 5).

**Figure 6:**
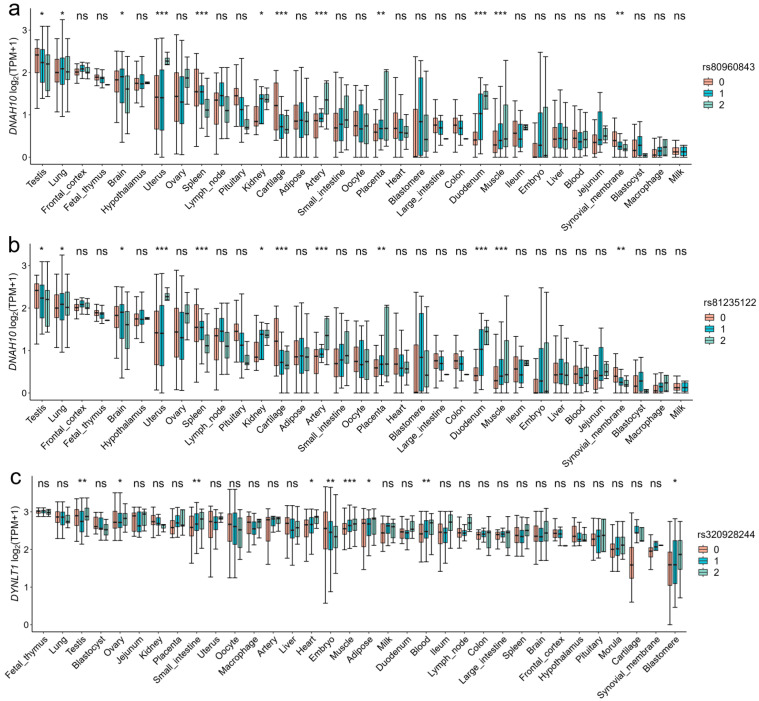
Effects of three genotypes of significant SNPs on gene expression levels in pig-specific tissues. (**a**,**b**) The distribution of the *DNAH10* gene expression for the significant SNPs (rs80960843 and rs81235122) across 34 tissues. (**c**) The distribution of the *DYNLT1* gene expression for the SNP rs320928244 across 34 tissues. The SNP rs320928244 has a high LD (R^2^ = 0.929) with the significant SNP rs80814693 of *DYNLT1*. The *y*-axis represents the gene expression levels, while the *x*-axis represents different tissues. Significant differences between genotypes (0, 1, and 2 represented genotypes AA, Aa, and aa, respectively) and genotype expression are obtained using the least significant difference. ns means *p* > 0.05, * means *p* < 0.05, ** means *p* < 0.01, *** means *p* < 0.001.

By calculating the TPM gene expression levels of candidate genes in 34 tissues, we found that the candidate genes *CCDC38*, *DNAH10*, *SBNO1*, *CCDC62*, *IFT81*, *SPATA6* and *GTSF1L* in Duroc, *DNAI2*, *METTL3*, *PNLDC1*, *RSPH3*, and *DYNLT1* in Landrace, and *RSPH3*, *DYNLT1*, *LHX9*, *PANX1*, *DHH*, and *CCDC65* in Yorkshire exhibited highly expression levels in testis tissue (Figure S5). The three genotypes of the SNPs rs81235122, rs80960843, and rs320928244 were also found to exhibit high expression levels in pig testis tissue. Furthermore, we found significant differences in the expression of the *DYNLT1* gene among the three genotypes of the SNP rs320928244 in pig testis tissue (Figure 6).

## 4. Discussion

In this study, we performed a weighted genome-wide association study for the semen traits and multi-tissue transcriptome analysis in three pig breeds. Among these, 5457 RNA-seq data containing multiple pig breeds were obtained from FarmGTEx databases. The PigGTEx Phase 1 (Pilot) relied on publicly available RNA-Seq and WGS datasets to establish a foundational resource for tissue-specific gene expression in pigs. Despite its significance, this phase was constrained by several data gaps, such as incomplete coverage across tissues and breeds, and variability in data quality among sources. To address these challenges, multiple strategies were employed. Normalization techniques were applied to RNA-Seq data to reduce inter-study variability, while missing genomic information was inferred through imputation methods based on high-quality reference panels. Where possible, supplementary datasets and insights from related species were incorporated to provide a more holistic perspective. These efforts were particularly impactful for tissues of the hypothalamus–pituitary–gonadal axis, where data reliability allowed for more detailed analyses of gene expression patterns critical to reproductive traits. However, the analysis was limited for tissues and breeds with lower data quality or availability, and these gaps were noted as areas for improvement in future phases. While these strategies helped mitigate biases, the limitations underscore the need for continued data collection and integration in subsequent PigGTEx initiatives to achieve a truly comprehensive tissue-specific atlas for pigs.

The variance components results indicated that semen traits were moderate heritabilities traits, similar to the finding of Marques et al. [6], and higher than those reported by Gao et al. [8]. Moderate heritability indicates that semen traits have large genetic variation and can be selected. Additionally, we found that the heritabilities of semen traits in Duroc were higher than those in Landrace and Yorkshire, except for SPPMOT, which was consistent with the findings of Li et al. [27]. Therefore, the selection strategies of semen traits in three breeds should be considered separately. Furthermore, the results of principal components analysis indicated the absence of outliers, possibly due to the introduction of boar populations from the same pasture, which possesses a homogenous genetic structure. This finding further reinforces the high quality and reliability of our samples.

Collecting a large-scale phenotypes dataset of semen traits and genotypes of boars is challenging due to the high cost involved. However, a potential solution to this problem is to implement weighted GWAS, which helps reduce stratification and stabilize the solution by assigning weights to the residual variance [12]. This method effectively decreases the standard errors of the model parameters and increases the power of the tests. In this study, the lambda value, which indicates the extent of population stratification, was taken into account and was found to be close to 1 (ranging from 0.89 to 1.09). This suggests that reasonable consideration is given to population stratification within the three breeds. One significant advantage of the weighted GWAS lies in the mitigation of confounding effects related to population structure, which can otherwise lead to false positives or negatives in GWAS.

The GWAS results revealed many hits seem to be singletons, the GWAS regions represented by only one associated SNP. This finding is consistent with the studies conducted by Hering et al. [26] and Mei et al. [10], who also encountered a similar situation. This may be due to limitations in the 50k chip data, leading to the detection of fewer SNPs associated with the target trait. To avoid false positives for single significant SNPs, we combined RNA-seq data to analyze gene expression levels of candidate genes found in significant SNPs GWAS regions. Interestingly, it was found that the identified candidate genes associated with semen traits were highly expressed in testis tissue. The presence of a single significant SNP in the GWAS results does not necessarily indicate unreliable. It may be attributed to the limited number of SNPs available in the 50 K chip data compared to whole genome sequencing data, as noted by Wang et al. [28]. Using whole-genome sequencing data is more conducive to detecting a higher proportion of significant SNPs in GWAS.

Several candidate genes have been reported to be associated with spermatogenesis, testicular function, and male fertility in pig semen traits. However, only a limited number of the same candidate genes have been identified across different studies. The majority of candidate genes associated with semen traits are specific to certain breeds or populations, potentially due to differences in the degree of selection for semen traits [6]. Additionally, the frequency of the same gene can vary between populations, leading to significant SNPs identified in GWAS results for one population not necessarily being significant in other populations [29]. Nevertheless, the utilization of similar analytical methods may play a crucial role in the low reproducibility observed across different studies.

In this study, the candidate genes *RSPH3* and *DYNLT1* were found to be located in the same region (8.24~8.56 Mb) on chromosome 1. *RSPH3* and *DYNLT* were identified as shared candidate genes of Landrace and Yorkshire, indicating a potential same QTL region between these two breeds. Mutations in the *RSPH3* gene have been associated with primary ciliary dyskinesia in humans, a disease characterized by defects in axial filaments in mobile cilia and sperm flagella [30]. *DYNLT1* encodes part of the motor complex and cytoplasmic dynamic protein and aberrant expression of *DYNLT1* has been linked to male infertility in humans [31]. Additionally, GO and KEGG enrichment analysis revealed that the candidate genes identified in three pig breeds were involved in shared biological processes, which is consistent with findings from another study [8].

For Duroc, seven candidate genes associated with semen traits have been reported to be linked to the biological function of sperm. One of the candidate genes, *CCDC38*, is located on chromosome 5: 87.56–87.61 Mb. The protein encoded by the *CCDC38* gene is considered a component of centromere protein in mammalian sperm cells and plays a crucial role in the nucleation of sperm ciliary axoneme [32]. *DNAH10*, located on chromosome 14 at 29.09–29.24 Mb, is in close proximity to *SBNO1*, located on chromosome 14 at 29.57–29.62 Mb. *DNAH10* gene is a component of the outer arm and inner arm dynamic protein attached to the peripheral microtubule duplex, specifically the inner arm dynamic protein heavy chain. Biallelic mutations in this gene can lead to primary male infertility in humans and mice with asthenoteratozoospermia [33]. The expression of the *SBNO1* gene is negatively correlated with human sperm motility [34]. Additionally, candidate genes *CCDC62* and *IFT81* are located on chromosome 14: 29.99–30.06 Mb and chromosome 14: 31.36–31.65 Mb, respectively. *CCDC62* is involved in the response of cells to estrogen stimulation and the positive regulation of transcription by RNA polymerase II, activating estrogen receptor binding activity and nuclear receptor coactivator activity. It is associated with spermatogenesis defects and male infertility [35,36]. The protein encoded by *IFT81*, together with *IFT74*, forms a module of the intraflagellar transport complex B, which is responsible for the transport of tubulin within the cilium. This protein plays a crucial role in spermatogenesis, fertility, and ciliogenesis [37,38]. Another candidate gene, *SPATA6*, located on chromosome 6: 163.27–163.40 Mb, was also identified in a study conducted by Marques et al. [6]. The *SPATA6* gene affects the expression of semen traits through biological processes such as cilium organization and assembly in mice and humans, particularly motile cilium assembly and spermatogenesis, which are active in sperm connecting pieces [39,40,41]. The *GTSF1L* is located on chromosome 17: 46.29–46.30 Mb and is specifically expressed in gonocytes and spermatids in mice [42].

For Landrace, five candidate genes associated with semen traits have been reported to be linked to the biological function of sperm. One of the candidate genes, *DNAI2*, *is* located on chromosome 12: 6.80–6.83 Mb and is part of the dynein complex of sperm flagella. It has been associated with sperm ciliary dyskinesia in humans [43], which is consistent with findings from studies conducted by Marques et al. [6] and Gao et al. [8]. The *DNAI2* gene is highly expressed in the human testis, and its mutation leads to primary ciliary motility disorders, resulting in impaired sperm flagellar function, leading to decreased fertility in men [44,45,46]. Another candidate gene, *METTL3*, is located on chromosome 7: 77.66–77.69 Mb and plays an important role in regulating spermatogenesis and the initiation of meiosis in mice [47]. The candidate gene *PNLDC1*, located on chromosome 1: 7.56–7.58 Mb, has been found in the endoplasmic reticulum and is implicated in spermatogenic failure. *PNLDC1* is necessary for meiosis and the development of male germ cells after meiosis in mice [48], affecting spermatogenesis and male fertility by modifying piRNA [49,50,51].

For Yorkshire, seven candidate genes associated with semen traits have been reported to be linked to the biological function of sperm. Among these candidate genes, *IZUMO1R* and *PANX1*, are located on chromosome 9: 26.44–26.52 Mb. *IZUMO1R* plays an important role in signal transduction during sperm–oocyte binding and the activation of sperm-binding site actin in mammals [52,53]. Mutations in this gene have been associated with in vitro fertilization failure in humans [54]. Homozygous variants in *PANX1* have been found to cause oocyte death and female infertility [55]. *LHX9* is located on chromosome 10: 20.74–20.76 Mb, belongs to the LIM homeobox gene family and encodes transcription factors involved in development. Inactivation of the *LHX9* gene leads to gonadal hypoplasia in mice, indicating its crucial role in gonadal development [56,57]. Furthermore, *DHH* and *CCDC65* are located on chromosome 5: 15.11–15.12 Mb and chromosome 5: 14.95–14.96 Mb, respectively. The *DHH* gene encodes a member of the Hedgehog family, which plays a critical role in regulating morphogenesis. Mutations in the *DHH* gene have been associated with partial gonadal hypoplasia with micronucleus polyneuropathy. *DHH* gene mutations affect male gonadal differentiation and neuromembrane development, with mild mutations impacting fertility and severe or multiple mutations leading to gonadal dysplasia [58,59,60]. The *CCDC65* gene affects semen traits through biological processes involving the axoneme and ciliary basal body. It encodes a sperm tail protein that is highly expressed in adult testis, spermatocytes, and sperm cells. *CCDC65* is a central hub for assembling nexin–dynein regulatory complexes, other cilia, and flagellar motility regulators in humans [61]. Mutations in *CCDC65* alter cilia beating patterns and cause primary ciliary dyskinesia in humans [62].

The integration of GWAS and RNA-seq data offers a robust framework to elucidate the genetic architecture underlying complex traits. In the study, data from weighted GWAS and multi-tissue RNA-seq were combined to identify genetic variants and candidate genes across three pig breeds. The methodology for integrating these datasets hinges on leveraging eQTL analyses, which assess the impact of genetic variants on gene expression in specific tissues. For calculating differences between breeds, tissues, and breed-by-tissue interactions, we employed a mixed model framework. Genotypic data were encoded (e.g., AA, Aa, and aa as 0, 1, and 2, respectively) and analyzed alongside phenotypic data, such as de-regressed estimated breeding values. The RNA-seq data, representing expression levels across 34 tissues, were used to calculate transcripts per million values, which were then mapped to significant SNPs identified in the GWAS. Breed differences were captured by stratifying analyses per breed (Duroc, Landrace, and Yorkshire), while tissue-specific effects were assessed by examining eQTLs associated with significant SNPs in RNA-seq data from specific tissues, such as the testis. For example, SNP rs320928244, significant in Landrace for sperm count traits, was found to regulate the expression of the *DYNLT1* gene specifically in testis tissue. This relationship was validated through linkage disequilibrium analysis and comparative genotype expression studies, revealing significant genotype-based expression differences. Similarly, for breed-by-tissue interactions, the study found overlapping eQTLs and significant SNPs for genes like *DNAH10* in testis tissues across breeds, emphasizing both shared and distinct genetic regulation mechanisms. This integration method not only highlights breed-specific and shared candidate genes but also provides insights into the biological pathways active in semen traits, facilitating improved breeding strategies.

## 5. Conclusions

This study integrated a weighted GWAS and multi-tissue transcriptome analysis to identify genetic variants and candidate genes associated with semen traits in three pig breeds. The findings revealed that most candidate genes were linked to sperm flagellar assembly, including components of axonemal dynein arms and microtubules. Mutations in these candidate genes were predominantly associated with sperm motility defects and male infertility. The multi-tissue transcriptome analysis indicated high expression levels of the identified candidate genes associated with semen traits in the testis tissue of Duroc, Landrace, and Yorkshire. Furthermore, significant effects of three genotypes of the rs320928244 on the *DYNLT1* gene expression in pig testis tissue. This study provides new insights into the genetic structure of semen traits in these three pig breeds, enabling the improvement of semen quality and yield through pig genome breeding.

## Figures and Tables

**Table 1:** Descriptive statistics and heritabilities of the semen traits for three pig breeds.

Traits ^a^	Breed	Number of Boars	Number of Records	Mean ± SD	Min	Max	$h^{2}$ (SE)	$r_{e}$ (SE)
SPMOT/%	Duroc	382	14,071	81.25 ^a^ ± 12.36	10.00	100.00	0.33 (0.06)	0.39 (0.02)
	Landrace	290	7826	75.42 ^b^ ± 15.92	10.00	100.00	0.20 (0.07)	0.33 (0.03)
	Yorkshire	264	6099	80.72 ^a^ ± 12.97	11.00	100.00	0.18 (0.04)	0.34 (0.02)
SPPMOT/%	Duroc	382	13,999	26.64 ^c^ ± 18.44	1.00	100.00	0.19 (0.04)	0.23 (0.02)
	Landrace	290	7793	38.85 ^b^ ± 21.19	1.00	100.00	0.20 (0.06)	0.23 (0.02)
	Yorkshire	264	6089	41.21 ^a^ ± 19.82	1.00	100.00	0.18 (0.07)	0.20 (0.02)
SPABR/%	Duroc	382	14,128	28.13 ^a^ ± 12.37	2.00	100.00	0.35 (0.08)	0.62 (0.02)
	Landrace	290	7860	20.49 ^b^ ± 13.80	1.00	100.00	0.27 (0.13)	0.71 (0.02)
	Yorkshire	264	6110	19.99 ^b^ ± 11.50	2.00	84.00	0.14 (0.09)	0.54 (0.02)
SPCOUNT/billions/mL	Duroc	382	13,877	34.97 ^b^ ± 18.85	5.00	177.90	0.25 (0.05)	0.33 (0.02)
	Landrace	290	7723	36.51 ^a^ ± 23.31	5.01	322.00	0.10 (0.05)	0.20 (0.02)
	Yorkshire	264	6051	33.29 ^c^ ± 20.33	5.02	294.80	0.14 (0.06)	0.20 (0.02)

^a^ SPMOT: sperm motility; SPPMOT: sperm progressive motility; SPABR: sperm abnormality rate; SPCOUNT: total sperm count/billions. In the same column, values with different letter superscripts mean significant difference (*p* < 0.05), while with same letter superscripts mean no significant difference (*p* > 0.05).

**Table 2:** The significant SNPs and candidate genes of semen traits in three pig breeds.

Breed	Trait ^a^	Chr	Position	RS Number	MAF	*p*-Value	FDR	gVar(%) ^b^	Number ^c^	Candidate Genes ^d^
Duroc	SPPMOT	5	87173753	rs325309529	0.36	3.11 × 10^−6^	0.0114	0.067	15	*CCDC38*
		5	87197703	rs80898749	0.35	1.12 × 10^−6^	0.0113	0.073	15	
		14	29085576	rs81235122	0.29	7.47 × 10^−6^	0.0190	0.074	32	*DNAH10*, *SBNO1*
		14	29522138	rs80960843	0.29	7.47 × 10^−6^	0.0190	0.074	37	*CCDC62*
		14	32290770	rs80896540	0.29	7.47 × 10^−6^	0.0190	0.074	25	*IFT81*
	SPABR	6	163993991	rs326805894	0.28	4.92 × 10^−6^	0.0479	0.083	18	*SPATA6*
		17	45749556	rs81466649	0.21	2.28 × 10^−6^	0.0422	0.088	10	*GTSF1L*
Landrace	SPABR	12	7629663	rs81243902	0.22	8.56 × 10^−6^	0.0413	0.198	14	*DNAI2*
		12	7655010	rs81437887	0.22	8.56 × 10^−6^	0.0413	0.198	13	
		12	7733404	rs81438684	0.22	8.56 × 10^−6^	0.0413	0.198	12	
	SPCOUNT	7	76901392	rs332051246	0.28	2.37 × 10^−6^	0.0360	0.069	21	*METTL3*
		1	7949224	rs80814693	0.33	1.76 × 10^−6^	0.0360	0.049	41	*PNLDC1, RSPH3*, *DYNLT1*
Yorkshire	SPMOT	1	9066284	rs81339403	0.18	6.41 × 10^−6^	0.0487	0.119	13	*RSPH3*, *DYNLT1*
		10	21117838	rs81422002	0.16	7.23 × 10^−7^	0.0180	0.154	7	*LHX9*
	SPABR	9	26895909	rs81408354	0.21	3.31 × 10^−9^	0.0001	0.151	19	*IZUMO1R*, *PANX1*
	SPCOUNT	5	14274707	rs81382568	0.15	4.51 × 10^−8^	0.0002	0.039	23	*DHH*, *CCDC65*

^a^ SPMOT: sperm motility; SPPMOT: sperm progressive motility; SPABR: sperm abnormality rate; SPCOUNT: total sperm count; ^b^ Percentage of genetic variance explained by significant SNPs; ^c^ The number of candidate genes associated with semen traits in a single SNP GWAS region; ^d^ The candidate gene(s) identified within searched regions.

**Table 3:** GO terms and KEGG pathways where the candidate genes were significantly (*p* < 0.05) enriched.

Term ^a^	Count	*p* Value	Candidate Genes
GO:0005813~centrosome	7	1.21 × 10^−5^	*CCDC38*, *IFT52*, *STIL*, *IFT81*, *CEP295*, *CCDC92*, *CCT5*
GO:0005814~centriole	5	5.06 × 10^−5^	*NEDD1*, *IFT52*, *STIL*, *CEP295*, *CCDC92*
GO:0007224~smoothened signaling pathway	4	2.97 × 10^−4^	*IFT52*, *STIL*, *DHH*, *TCTN2*
GO:0005930~axoneme	3	4.61 × 10^−4^	*DNAI2*, *DNAH10*, *CCDC65*
GO:0008233~peptidase activity	2	0.0016	*DHH*, *USP44*
GO:0036064~ciliary basal body	4	0.0017	*NEDD1*, *IFT52*, *IFT81*, *CCDC65*
GO:0060271~cilium assembly	4	0.0044	*IFT52*, *IFT81*, *TCTN2*, *TCTN1*
GO:0005929~cilium	3	0.0218	*IFT52*, *DNAH10*, *IFT81*
GO:0007342~fusion of sperm to the egg plasma membrane	2	0.0355	*IZUMO1R*, *LLCFC1*
ssc05014: amyotrophic lateral sclerosis	3	0.0035	*DNAI2*, *ATXN2*, *DNAH10*

^a^ GO, Gene Ontology; KEGG, Kyoto Encyclopedia of Genes and Genomes pathway.

## Data Availability

The datasets generated and/or analyzed during the current study are not publicly available since the studied population consists of the nucleus herd of Guangdong Guyue Technology Co., Ltd., China, but are available from the corresponding author on reasonable request.

## Supplementary Materials

The following supporting information can be downloaded at: https://www.mdpi.com/article/10.3390/ani15030438/s1, Figure S1: The distribution histogram with semen collection times of Duroc, Landrace, and Yorkshire; Figure S2: The distributions histogram of DEBVs phenotype for semen traits in three pig breeds; Figure S3: Quantile–quantile plots for GWAS results of semen traits in the Duroc, Landrace, and Yorkshire; Figure S4: The 34 tissues sample size of 5457 pig RNA-seq data from FarmGTEx; Figure S5: The expression levels of candidate genes associated with semen traits in 34 tissues; Table S1: Variance components of the semen traits of three pig breeds; Table S2: Descriptive statistics of DEBVs for the semen traits in three pig breeds; Table S3: All candidate genes in GWAS regions of semen traits in three pig breeds.
