# Supplementary material for: Identified Candidate Genes of Semen Trait in Three Pig Breeds Through Weighted GWAS and Multi-Tissue Transcriptome Analysis
Source: Animals (Basel). 2025 Feb 5;15(3):438. doi: 10.3390/ani15030438 (PMC11816172; doi:10.3390/ani15030438)
Supplement: Supplementary file 1 [file animals-15-00438-s001.zip › animals-3399835-supplementary.pdf]

Supplementary Materials

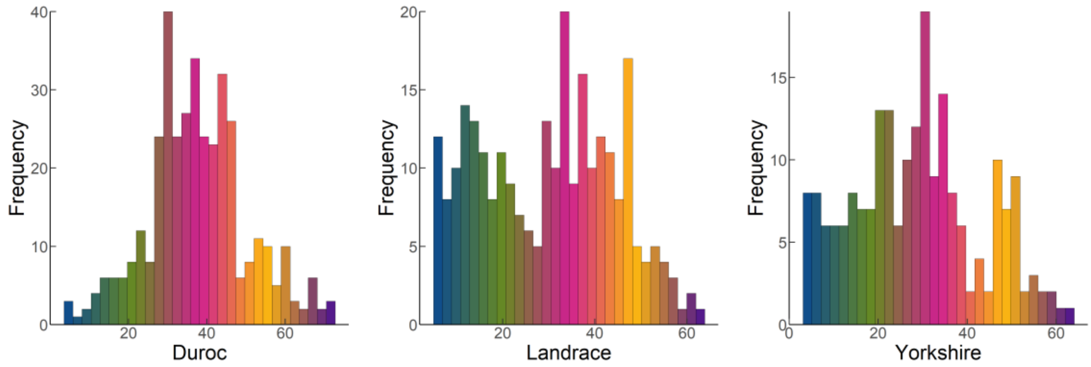

Figure S1. The distribution histogram with semen collection times of Duroc, Landrace, and Yorkshire.

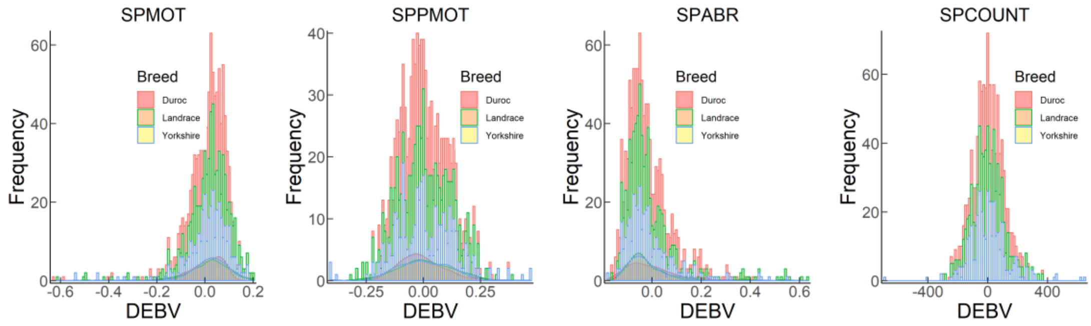

Figure S2. The distributions histogram of DEBV phenotype for semen traits in three pig breeds.

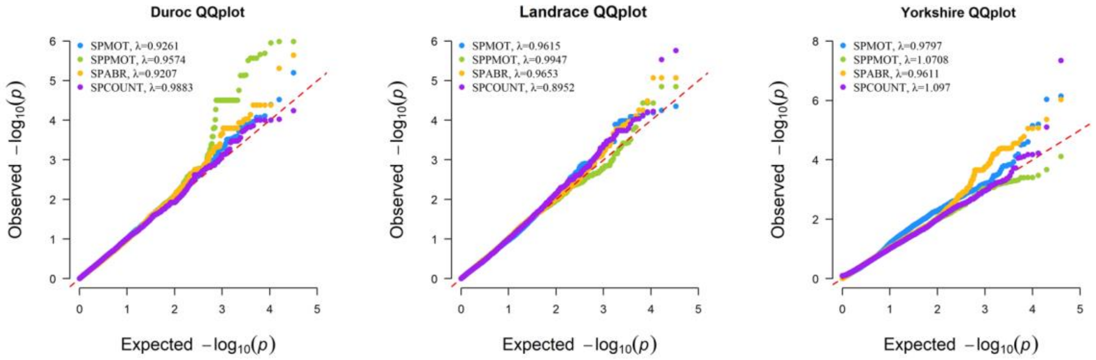

Figure S3. Quantile–quantile plots for GWAS results of semen traits in the Duroc, Landrace, and Yorkshire.

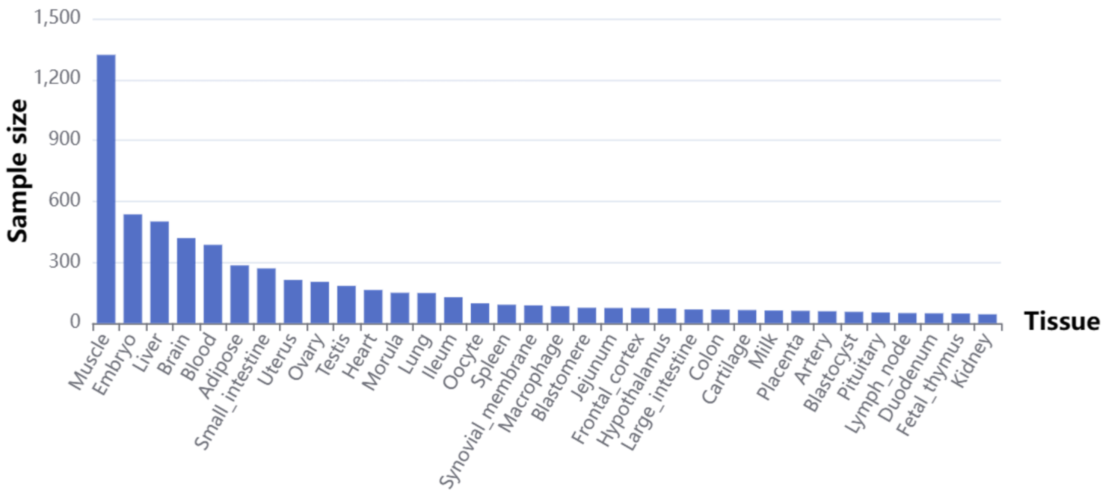

Figure S4. The 34 tissues sample size of 5,457 pig RNA-seq data from FarmGTEx.

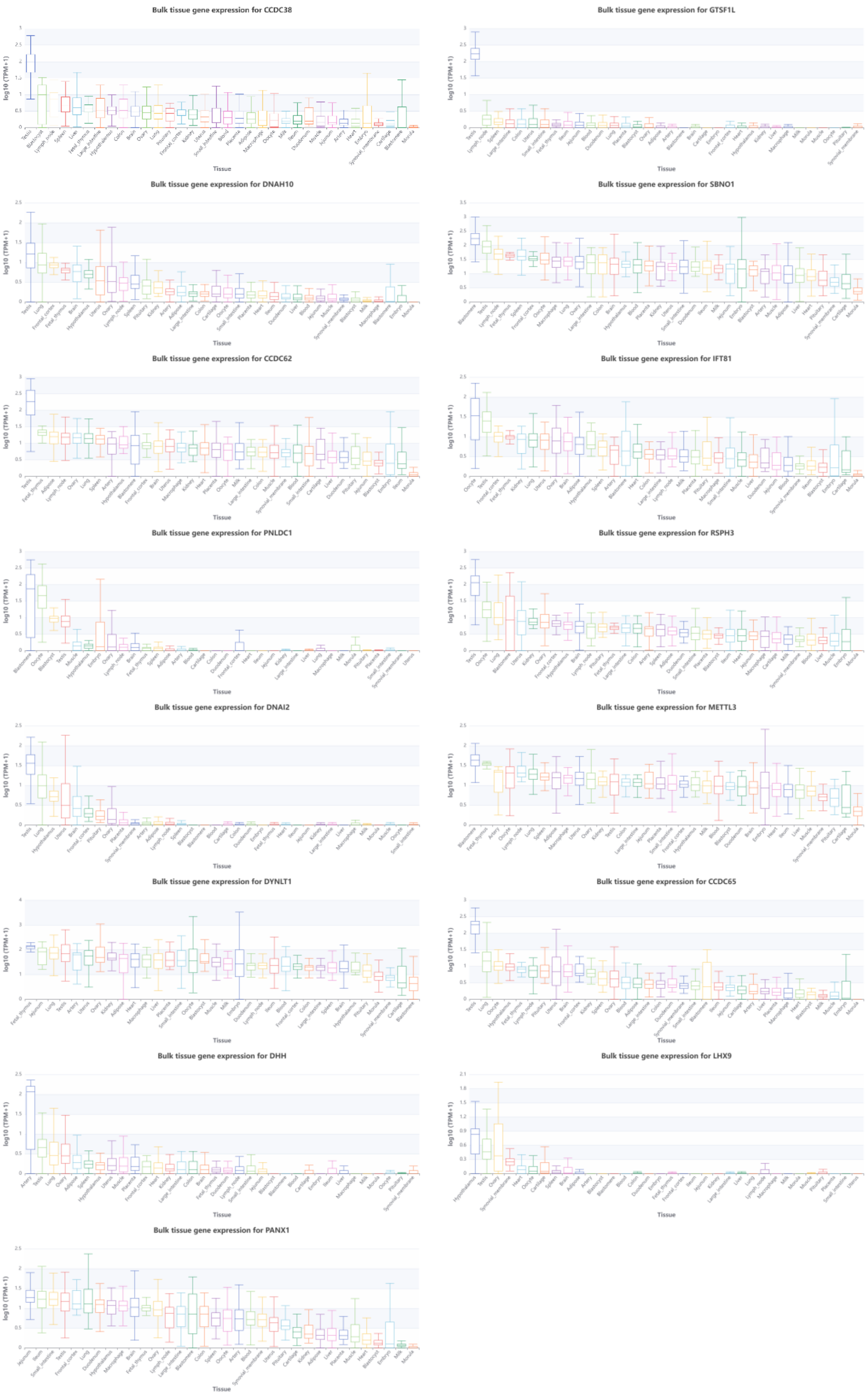

Figure S5. The expression levels of candidate genes associated with semen traits in 34 tissues.

Table S1. Variance components of the semen traits of three pig breeds

Trait	Breed	σ_a^2(SE)	σ_p^2(SE)	σ_e^2(SE)	h^2(SE)	r_e(SE)
SPMOT	Duroc	59.19(12.91)	12.05(8.33)	110.75(1.07)	0.33(0.06)	0.39(0.02)
	Landrace	53.14(20.36)	36.99(14.53)	179.41(2.59)	0.20(0.07)	0.33(0.03)
	Yorkshire	41.75(5.87)	35.46(6.29)	150.23(1.90)	0.18(0.04)	0.34(0.02)
SPPMOT	Duroc	61.47(14.52)	12.48(9.44)	252.27(2.43)	0.19(0.04)	0.23(0.02)
	Landrace	86.65(28.78)	15.20(18.40)	332.98(4.82)	0.20(0.06)	0.23(0.02)
	Yorkshire	66.98(26.04)	8.56(18.60)	298.78(4.90)	0.18(0.07)	0.20(0.02)
SPABR	Duroc	64.99(17.77)	49.82(12.45)	71.76(0.69)	0.35(0.08)	0.62(0.02)
	Landrace	63.35(32.32)	100.57(25.23)	67.95(0.98)	0.27(0.13)	0.71(0.02)
	Yorkshire	20.34(13.63)	59.31(12.39)	67.49(1.11)	0.14(0.09)	0.54(0.02)
SPCOUNT	Duroc	8318.26(1887.56)	2419.43(1235.51)	21923.87(212.31)	0.25(0.05)	0.33(0.02)
	Landrace	5233.98(2455.11)	4590.52(1811.66)	40278.63(585.23)	0.10(0.05)	0.20(0.02)
	Yorkshire	4977.66(2080.75)	2278.47(1531.54)	28525.49(469.00)	0.14(0.06)	0.20(0.02)

Table S2. Descriptive statistics of DEBVs for the semen traits in three pig breeds

Trait	Breed	Number of boars	Mean[2]	SD[3]	Max[4]	Min[5]
SPMOT	Duroc	361	0.0006893	0.092	0.139	-0.631
	Yorkshire	257	0.0000546	0.114	0.201	-0.614
	Landrace	232	0.0113090	0.094	0.179	-0.537
SPPMOT	Duroc	361	0.0083109	0.098	0.373	-0.224
	Yorkshire	257	0.0062132	0.121	0.388	-0.318
	Landrace	232	0.0027516	0.127	0.466	-0.409
SPABR	Duroc	361	0.0000081	0.108	0.431	-0.165
	Yorkshire	257	0.0058780	0.140	0.633	-0.163
	Landrace	232	0.0105506	0.108	0.554	-0.182
SPCOUNT	Duroc	361	6.0531191	104.541	359.464	-251.933
	Yorkshire	257	1.2429568	115.542	371.399	-303.441
	Landrace	232	6.0102260	137.470	645.794	-691.845

Table S3. All candidate genes in GWAS regions of semen traits in three pig breeds

Breed	Traits	SNP[a]	Count[b]	Candidate genes
Duroc	SPMOT	8_130866268	17	*TIGD2, FAM13A, NAP1L5, HERC5, HERC6, PPM1K, ABCG2, PKD2, SPP1, MEPE, IBSP, DMP1, DSPP, SPARCL1, HSD17B11, KLHL8, AFF1*
	SPPMOT	5_86255932, 5_86771075, 5_86839675, 5_87173753, 5_87197703, 14_27217553, 14_27251927, 14_27928204, 14_27973397, 14_28094509, 14_29085576, 14_29522138, 14_32290770	90	*MEM132C, AACS, BRI3BP, DHX37, UBC, SCARB1, NCOR2, RFLNA, ZNF664, CCDC92, DNAH10, ATP6V0A2, SUMO4, TCTN2, EIF2B1, GTF2H3, DDX55, RILPL1, SNRNP35, RILPL2, KMT5A, SBNO1, CDK2AP1, MTRFR, MPHOSPH9, PITPNM2, ARL6IP4, OGFOD2, ABCB9, VPS37B, HIP1R, CCDC62, DENR, HCAR1, KNTC1, RSRC2, ZCCHC8, CLIP1, DIABLO, B3GNT4, ANAPC5, CAMKK2, P2RX4, IFT81, P2RX7, ATP2A2, ANAPC7, ARPC3, GPN3, FAM216A, VPS29, RAD9B, PPTC7, TCTN1, HVCN1, PPP1CC, MYL2, CUX2, PHETA1, SH2B3, ATXN2, BRAP, ACAD10, CIT, PRKAB1, CCDC60, APAF1, IKBIP, SLC25A3, SNORA53, TMPO, ssc-mir-135-2, MIR1251, Metazoa, SRP, NEDD1, CDK17, ELK3, SNORA72, LTA4H, HAL, AMDHD1, CCDC38, SNRPF, NTN4, USP44, METAP2, ssc-mir-331, VEZT, FGD6*
	SPABR	6_163993991, 17_45749556	29	*AGBL4, SPATA6, SLC5A9, TRABD2B, FOXD2, FOXE3, CMPK1, STIL, TAL1, CYP4A24, CYP4B1, EFCAB14, SNORA73, TEX38, ATPAF1, MOB3C, MKNK1, KNCN, DMBX1, PTPRT, SRSF6, SGK2, IFT52, MYBL2, GTSF1L, TOX2, OSER1, GDAP1L1, FITM2*
	SPCOUNT	-	-	-
Landrace	SPMOT	-	-	-
	SPPMOT	-	-	-
	SPABR	12_7629663, 12_7655010, 12_7733404, 13_136995166, 13_137134779, 16_3483525	35	*CD300C, CD300LB, GPRC5C, GPR142, BTBD17, KIF19, DNAI2, TTYH2, RPL38, CDC42EP4, C17orf80, FAM104A, COG1, SLC39A11, SSTR2, SOX9, CCDC14, MYLK, HACD2, ADCY5, SEC22A, PDIA5, SEMA5B, SLC49A4, HSPBAP1, PARP14, DTX3L, PARP9, KPNA1, FAM162A, DNAH5, TRIO, OTULINL, OTULIN, ANKH*
	SPCOUNT	1_7949224, 7_27310755, 7_76901392	62	*MAS1, MAP3K4, PLG, SLC22A3, SLC22A2, SLC22A1, IGF2R, PNLDC1, MRPL18, TCP1, ACAT2, WTAP, SOD2, FNDC1, TAGAP, RSPH3, EZR, SYTL3, DYNLT1, TMEM181, SERAC1, TINAG, MLIP, LRRC1, KLHL31, GCLC, KHDRBS2, PRIM2, TRAV6, TRAV2, OR6J1, OR4E1, OR10G3, SLC7A8, CEBPE, C14orf119, ACIN1, OR4E2, CDH24, C14orf93, AJUBA, PRMT5, PSMB11, RBM23, REM2, SLC7A7, LRP10, MMP14, MRPL52, OXA1L, ABHD4, DAD1, TRDC, TRAV36DV7, TRAV16, TRAV4, TRAV3, SALL2, METTL3, CHD8, SUPT16H, RPGRIP1*
Yorkshire	SPMOT	1_9066284, 1_10247485, 5_15240264, 10_21117838	32	*FNDC1, TAGAP, RSPH3, EZR, SYTL3, DYNLT1, TMEM181, SERAC1, SYNJ2, SNX9, ZDHHC14, TMEM242, ARID1B, ssc-mir-9857, ssc-mir-9787, ATP2B1, POC1B, DUSP6, KITLG, TMTC3, CEP290, C12orf29, C12orf50, ssc-mir-181a-1, ssc-mir-181b-1, CRB1, DENND1B, C1orf53, LHX9, NEK7, ATP6V1G3, PTPRC*
	SPPMOT	-	-	-

	SPABR	7_28200326, 7_28205479, 9_26895909, 14_177223, 16_72533983, 18_6978182	91	*SNORA72, GCLC, KHDRBS2, PRIM2, RAB23, BAG2, ZNF451, BEND6, DST, ssc-mir-7143, CEP295, TAF1D, C11orf54, VSTM5, HEPHL1, PANX1, IZUMO1R, GPR83, MRE11, ANKRD49, C11orf97, PIWIL4, AMOTL1, CWC15, ENDOD1, SESN3, FAM76B, CEP57, MTMR2, SPIN1, NXNL2, S1PR3, SHC3, CKS2, SECISBP2, SEMA4D, GADD45G, SLC36A1, SLC36A2, SLC36A3, GM2A, CCDC69, ANXA6, TNIP1, GPX3, MARCHF6, CMBL, CCT5, ATPSCKMT, SEMA5A, TRBV25-1, TRBV3-1, MIR671, SMARCD3, CHPF2, H2BE1, IQCA1L, ASB10, GBX1, AGAP3, TMUB1, TAS2R41, FASTK, SLC4A2, ABCB8, ASIC3, ATG9B, KCNH2, AOC1, TMEM176A, GIMAP2, GIMAP4, GIMAP8, TCAF1, EPHA1, ZYX, FAM131B, TMEM139, GSTK1, TRPV5, KEL, LLCFC1, EPHB6, PRSS2, TRBV27, TRBV19, PRSS58, MGAM2, MGAM, TAS2R39, OR6V1*
	SPCOUNT	5_14274707, 6_10520551	29	*CRY1, MTERF2, TMEM263, RIC8B, POLR3B, TCP11L2, CKAP4, NUAK1, CCNT1, TEX49, ADCY6, CACNB3, DDX23, RND1, CCDC65, WNT10B, WNT1, DDN, PRKAG1, RHEBL1, DHH, LMBR1L, TUBA1B, WWOX, CLEC3A, VAT1L, NUDT7, ADAMTS18, MON1B*

[a] Significant SNPs, the SNP name is indicated by chr: position; [b] The number of candidate genes.
